# Giant bladder diverticulum in a postmenopausal woman: Case report and literature review

**DOI:** 10.1016/j.eucr.2021.101807

**Published:** 2021-08-12

**Authors:** Ousmane Sow, Alioune Sarr, Cyrille Ze Ondo, Babacar Sine, Abdoulaye Ndiath, Alain Khassim Ndoye

**Affiliations:** Urology-Andrology Department, Aristide Le Dantec Hospital, Dakar, Senegal

**Keywords:** Bladder, Diverticulum, Woman, Urinary retention

## Abstract

Bladder diverticulum represents a herniation of the bladder mucosa and submucosa through a point of weakness in the detrusor muscle. Bladder diverticula are rare and most often described in men. We report a symptomatic giant bladder diverticulum in a 56-year-old postmenopausal woman. The symptomatology was marked by acute urinary retention associated with abdominal-pelvic pain. A diverticulectomy by a transvesical approach with placement of a urethral catheter was performed. The postoperative course was uneventful.

## 1 Introduction

Bladder diverticulum is a result of the bladder mucosa and submucosa herniation through the muscularis propria of bladder wall^1^**.** Most cases are asymptomatic and discovered incidentally during an imaging study. Bladder diverticula can be congenital or acquired^1^**.** A giant diverticulum may manifest with symptoms such as urinary retention, urinary tract infections, hematuria, neoplasm formation, or even acute abdomen due to rupture^2^**.** We report a symptomatic giant bladder diverticulum in a postmenopausal woman. Through this case and a review of the literature, we will discuss the epidemiological, diagnostic and therapeutic aspects.

## 2 Case presentation

A 56- year -old postmenopausal patient, without any particular pathological history who consulted for an acute urinary retention having required the placement of a urethral catheter. This symptomalogy was preceded by lower urinary tract involving urinary frequency, dysuria lasting for about five months associated with abdominal-pelvic pains with an abdominal distension. Physical examination showed a good general condition, hypogastric sensitivity, the urethral meatus was not stenosed. Urine culture showed a urinary infection with *Escherichia coli* sensitive to ceftriaxon.

**Fig. 1:**
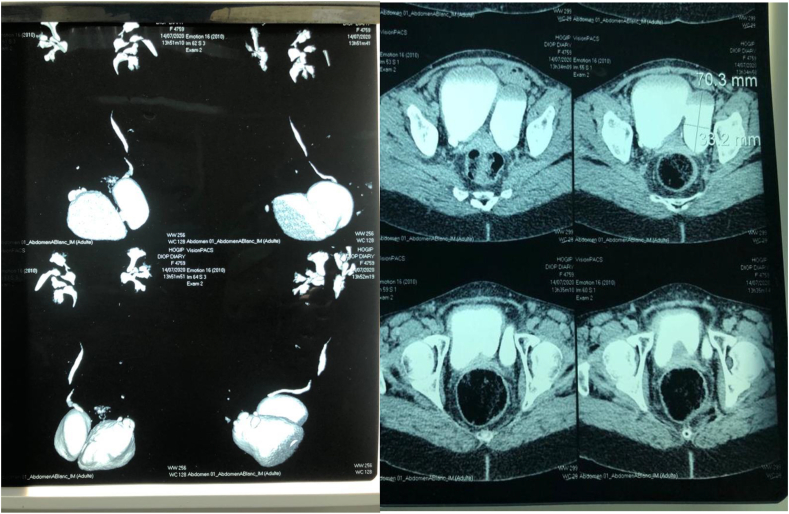
Computed tomography (CT) scan of the abdomen and pelvis showed a distended. Bladder with a thickened and irregular wall measuring up to 1 cm with the presence of a 70.3 × 33.2 mm diverticulum located at the level of the dome.

Abdominal and pelvic ultrasound revealed a thick-walled globular bladder and a left lateralvesical cystic formation of 134 × 130 mm, suggesting a left ovarian cyst. A computer tomography (CT) scan of the abdomen and pelvis showed a distended bladder with a thickened and irregular wall measuring up to 1 cm with the presence of a 70.3 × 33.2 mm diverticulum located at the level of the dome **(**Fig. 1**).** Urodynamic studies were not performed because of the insufficient medical facilities. The diagnosis of a symptomatic giant bladder diverticulum complicated by an acute urinary retention was retained.

**Fig. 2:**
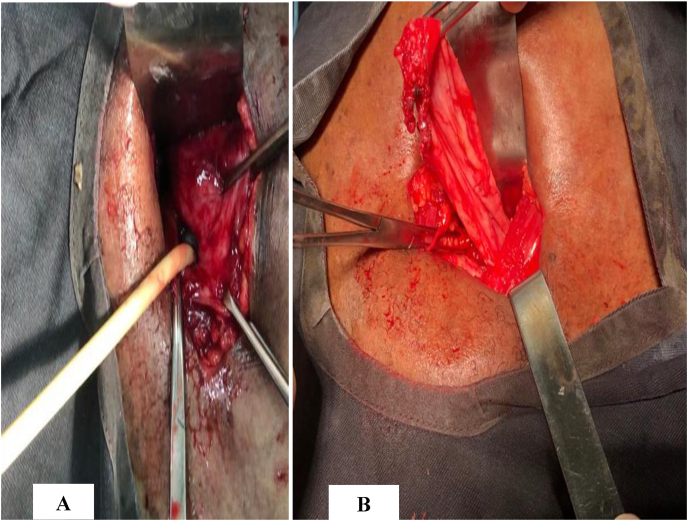
**(A)** Location of the diverticulum neck **(B)** Dissection of the diverticulum.

**Fig. 3:**
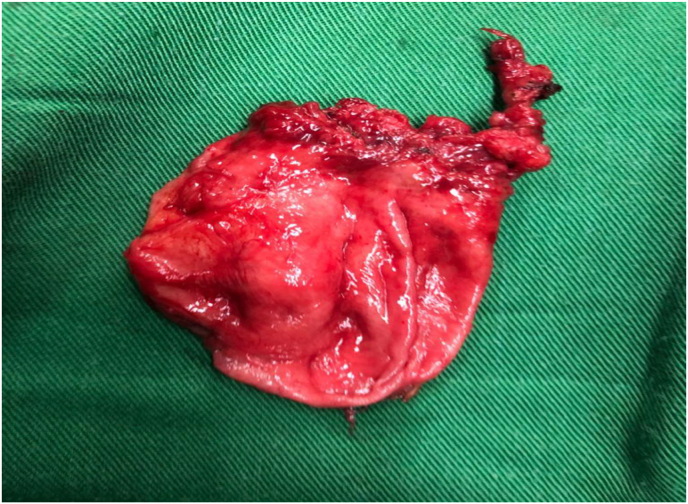
Complete resection of the diverticulum.

The patient was managed with ceftriaxon 2g/day in two doses for 3 days preoperatively and 7 days postoperatively. A diverticulectomy by a transvesical approach was performed. It allowed the location of the neck **(**Fig. 2 A**)** and the dissection of the diverticulum **(**Fig. 2B**)**. A complete resection of the diverticulum was performed with the placement of a urethral catheter **(**Fig. 3**)**. The removal of the catheter was done at 6 days post-operation and the postoperative course were uneventful. After a 6-month follow-up, the ultrasound scan of the urinary tract was unremarkable (no post-void residue, no diverticulum). However, the patient still reported lower urinary tract symptoms dominated by irritative signs.

## 3 Discussion

Bladder diverticula may be acquired or congenital^1^**.** Congenital bladder diverticula are believed to be due to a congenital weakness in the bladder wall musculature especially in the fibromuscular network near the urethral hiatus, which is usually present during childhood^1^**.**

Acquired diverticula are usually multiple. The most common acquired diverticula are believed to be the result of increased intravesical pressure caused by lower urinary tract obstruction, such as neurogenic bladder dysfunction or external sphincter dyssynergia.^2^ Bladder diverticula are rare and most often described in men^3^**.** Bladder diverticula are usually asymptomatic and discovered incidentally. The symptomatology is often atypical which leads to a delay in diagnosis. Bladder diverticula may manifest as dysuria such as incomplete voiding and straining, or urinary incontinence^1^**.** However, urinary tract infection secondary to post-void residual is the most common mode of manifestation for large diverticula^1^**.** Acute urinary retention complicating a bladder diverticulum is extremely rare^2^**.** Urine retention is due to the compression that the diverticulum may exert on the bladder neck^2^**.** Vesico-ureteral reflux that may be responsible for irreversible kidney damage is often associated^1^**.** In our case, acute urinary retention associated with abdominal, pelvic-pains revealed a giant bladder diverticulum, and there was no associated vesico-ureteral reflux.

Diagnosis was based on imaging, including ultrasonography, retrograde urethrocystography, Computed tomography scan and a cystoscopy. Ultrasound shows the diverticulum, a post-void residual, associated vesico-renal reflux or upper urinary tract involvement and appreciates the subvesical obstruction^4^**.** However, the ultrasonographic characteristics of the bladder diverticulum often seem similar to those of an ovarian cyst, as in our patient's case. Retrograde urethrocystography is the examination of choice for the detection of bladder diverticula^4^**.**

Computed tomography (CT) scan of the urinary tract is optional, but gives a more precise measurement of the volume of the diverticulum, appreciates the possible impact on the upper urinary tract and specifies the close relationship between the posterior surface of the diverticulum and the neighboring organs (rectum, and homolateral ureter)^4^**.** Cystoscopy specifies the dimensions of the neck of the diverticulum and its position in relation to the ureteral meatus. In some cases, a very wide neck can be crossed by the cystoscope, which allows visualization of the diverticulum wall and identification of a possible intra-diverticular ureteral orifice. Urodynamic examination, by recording physical parameters such as flow rates, pressures and volumes, provides additional information that is very useful in the etiological investigation^4^**.** Having eliminated any organic obstruction in our patient, a functional etiology seems more than plausible. A complete urodynamic study would have allowed us to determine the cause.

Diverticulectomy is the procedure of choice and can be performed by an extravesical, intravesical or combined approach with good results^1^**.** Laparoscopic and transurethral approaches are also available. Because the diverticulum is usually adherent to adjacent structures, such as the rectum and ureter, careful extravesical dissection during excision is imperative to avoid injury. The basic principle is to dissect close to the wall of the diverticulum and the bladder muscle defect must be meticulously repaired. In most cases, there is associated reflux or the ureteral meatus is involved by the diverticulum, making homolateral ureteral reimplantation necessary, but in cases where bilateral reflux is observed, then bilateral ureteral reimplantation is advised^5^**.** Our patient had a giant bladder diverticulum and presented with lower urinary tract disorders. We operated on our patient using an intravesical approach. The postoperative course was simple.

## 4 Conclusion

Giant bladder diverticulum is rare in women and the symptomatology is often non-specific. Urine retention is a rare complication of this condition. Surgical treatment is based on diverticulectomy often associated with ureteral reimplantation.

## Consent

Informed consent was provided by the patient.

## Funding sources

This research did not receive any specific grant from funding agencies in the public, commercial, or not-for-profit sectors.

## Authors' contributions

OS, A S and CZO: Drafting manuscript and bibliographic research through a review of the literature. B S, A N and AKN: Correction and elaboration of the final manuscript. They constituted the ethics committee that approved the document. All the authors have read and agreed to the final version of the manuscript.

## Declaration of competing interest

The authors have no conflicts of interest to declare.
